# The Effect of the Brain Breaks Physical Activity Program on Task Focus, Attentional Control, and Academic Achievement in 4th Grade Elementary School Students

**DOI:** 10.3390/bs16050804

**Published:** 2026-05-18

**Authors:** Bijen Filiz, Yasin Karaca, Ferman Konukman, Andrew Sortwell

**Affiliations:** 1 Department of Coaching Education, Afyon Kocatepe University, Afyonkarahisar 03200, Türkiye; bijenfiliz@aku.edu.tr; 2 Department of Recreation Management, Osmaniye Korkut Ata University, Osmaniye 80000, Türkiye; yasinkaraca@osmaniye.edu.tr; 3 The College of Sport Sciences, Qatar University, Doha 2713, Qatar; 4 School of Education, The University of Notre Dame Australia, Sydney 2007, Australia; andrew.sortwell@nd.edu.au; 5 School of Health Sciences, The University of Notre Dame Australia, Sydney 2007, Australia; 6 Research Centre in Sports Sciences, Health Sciences and Human Development (CIDESD), University of Beira Interior, 6201-001 Covilhã, Portugal

**Keywords:** Brain Breaks, classroom physical activity, attentional control, on-task behavior, classroom engagement, academic performance, elementary education

## Abstract

This study examined the effects of Brain Breaks (BB) physical activity videos on fourth-grade students’ on-task behavior, attentional control, and short-term academic achievement. A quasi-experimental pre-test/post-test control group design was employed with 49 students from a private school in Türkiye (experimental n = 24; control n = 25). Over four weeks, the experimental group participated in 48 BB sessions integrated into classroom instruction. Data collection included minute-by-minute observations of on-task behavior, the Attentional Control Scale for Children, and brief quizzes in Mathematics and Social Studies. Data were analyzed using repeated-measures ANOVA and paired sample *t*-tests. Descriptive analyses of on-task behavior revealed consistently higher initial engagement following BB sessions, particularly within the first few minutes of classroom activities. A significant Time × Group interaction was observed for attentional control, indicating greater improvement in the experimental group over time. In terms of academic outcomes, Social Studies scores increased significantly following the intervention, whereas no significant change was observed in Mathematics scores. Overall, the findings suggest that BB activities may support short-term attentional engagement and attentional control, with subject-specific effects on academic performance. These results highlight the potential of BB as a practical classroom strategy to enhance students’ readiness to engage in learning tasks.

## 1. Introduction

The widespread problem of prolonged sedentary behavior in educational settings is recognized as a significant contributor to adverse health outcomes and reduced cognitive performance in school-aged children (Hillman et al., 2019). Extended periods of sitting during classroom instruction may negatively affect attention, focus, and academic performance (Dong & Shen, 2026). Considering that children spend a substantial portion of their school time engaged in sedentary activities, integrating physical activity (PA) into classroom environments has become increasingly important for supporting both cognitive and developmental outcomes (Oliveira et al., 2023).

A growing body of research indicates that even brief bouts of PA can lead to immediate improvements in attentional performance and cognitive engagement (Wachira et al., 2022). Short active breaks may improve neurocognitive functioning and help students sustain attention during instructional time (Reyes-Amigo et al., 2025). Classroom-based PA interventions have been shown to enhance on-task behavior and attentional control, both of which are critical components of effective learning (Szabo-Reed et al., 2017). Similarly, recent research has demonstrated that structured Brain Break (BB)-type interventions, such as GoNoodle activities, can produce both acute and chronic improvements in academic-related outcomes, including reading fluency among elementary school students (Wold et al., 2023).

The cognitive benefits of PA are often explained through physiological and neurobiological mechanisms. Short-duration PA may increase cerebral blood flow and oxygenation in brain regions associated with memory and learning, while also enhancing neurotransmitter activity linked to attention and executive functioning (Syaukani et al., 2023). These changes may contribute to improved processing speed, concentration, and overall cognitive readiness. Furthermore, classroom-based PA breaks have been associated with immediate improvements in executive functioning and psychological well-being (Graham et al., 2021). Given these benefits, integrating PA into classroom routines represents a practical and cost-effective strategy for supporting student engagement (Peiris et al., 2022).

In this context, BB represents a structured form of classroom-based PA delivered through short, video-guided movement activities. Typically lasting 3–5 min, these activities combine elements such as coordination, rhythm, and interactive movement, making them both engaging and accessible within classroom settings. Previous research suggests that BB interventions can positively influence students’ attitudes toward PA and promote engagement in classroom tasks by reactivating attention following periods of sedentary instruction (e.g., Bonnema et al., 2022; Mok et al., 2020; Podnar et al., 2018).

Despite the growing evidence supporting the benefits of PA in educational settings, several limitations remain in the existing literature. Most studies have relied on pre–post designs or aggregated outcome measures, providing limited insight into how attention and engagement evolve during classroom activities (Schmidt et al., 2016; Szabo-Reed et al., 2017). In particular, the temporal dynamics of student engagement immediately following PA interventions remain underexplored (Mazzoli et al., 2021). Moreover, relatively few studies have examined how short bouts of PA influence moment-to-moment changes in on-task behavior within classroom contexts (Song et al., 2022).

The present study addresses these gaps by examining the effects of a four-week BB intervention on fourth-grade students’ on-task behavior, attentional control, and academic performance. In contrast to previous research, this study provides a fine-grained analysis of classroom engagement by investigating minute-by-minute changes in on-task behavior across multiple lessons. By focusing on the temporal dynamics of attention, the study aims to offer a more detailed understanding of how brief PA interventions influence students’ immediate readiness to engage in learning tasks. In doing so, it contributes to the literature by moving beyond aggregated measures and highlighting the time-sensitive nature of attention in classroom settings. This study addresses the following research questions:

1. Does the BB intervention lead to changes in students’ attentional control over time, and do these changes differ between the experimental and control groups?
2. How does students’ on-task behavior change over time during classroom instruction before and after BB sessions, based on minute-by-minute observations?
3. Does participation in BB activities lead to changes in short-term academic performance (Mathematics and Social Studies quiz scores) within the experimental group?

## 2. Materials and Methods

### 2.1. Study Design

A quasi-experimental pre-test/post-test control group design was employed, involving 49 fourth-grade students, to evaluate the effects of the BB intervention on various academic and cognitive outcomes. Existing classroom structures were maintained, and participants could not be randomly assigned individually to the experimental and control groups. Therefore, the study was conducted using a nonequivalent groups quasi-experimental design. During the implementation process, one classroom was assigned as the experimental group and the other as the control group. Pre-tests were administered to both groups before the intervention, and post-tests were administered after the intervention. The use of a quasi-experimental design was deemed appropriate given the logistical challenges of randomizing individual students in an intact classroom environment (Bartholomew et al., 2018). However, the absence of full randomization inherently introduces potential threats to internal validity, such as selection bias, which necessitates careful consideration during data interpretation. Therefore, efforts were made to ensure comparability between groups at baseline through pre-test measures and covariate analyses to mitigate these potential biases.

### 2.2. Participants

The study sample consisted of 49 fourth-grade students (aged 9–10 years) enrolled in a private college in Türkiye who had no prior experience with the BB PA program. Class sizes were typical for private institutions, and participants were recruited using convenience sampling, a purposive non-probability method based on voluntary participation and accessibility (Yıldırım & Şimşek, 2013). This approach was chosen for its practicality and efficiency, as convenience sampling allows researchers to select readily available participants, thereby enhancing the speed and feasibility of the study (Yıldırım & Şimşek, 2013). A priori power analysis (power = 0.80, α = 0.05, medium effect size) indicated that a minimum sample of 44 participants was required; thus, the final sample of 49 met recommended statistical power thresholds.

The experimental group comprised 24 students, and the control group comprised 25 students, drawn from two intact fourth-grade classes (Class 4-A as experimental and Class 4-C as control) that were equivalent in baseline attentional control levels (pre-test scores compared and matched to ensure similarity). During the experimental phase, the experimental group received the BB-focused PA intervention, while the control group continued with the standard curriculum without any additional PA breaks.

The study was conducted in a private primary school located in western Türkiye. The school follows the national curriculum and serves students primarily from middle- to upper-socioeconomic backgrounds.

### 2.3. Measures

#### 2.3.1. The Attentional Control Scale for Children (ACS-C)

This scale was originally developed by Derryberry and Reed (2002) and later adapted into Turkish by Akın et al. (2013). It consists of 20 items with a 4-point Likert-type response format (1 = Almost never–4 = Almost always). The total score derived from the items is considered an indicator of the individual’s attentional control level. The scoring range varies between 20 and 80, with higher scores assumed to reflect a higher level of attentional control. In this study, the internal consistency reliability of the scale was calculated using Cronbach’s alpha coefficient, which was found to be 0.86. This value indicates that the scale possesses an acceptable level of reliability (Tavşancıl, 2019).

#### 2.3.2. Mathematics and Social Studies Short Achievement Tests (Quizzes)

Short standardized quizzes consisting of 10–12 set questions, developed by curriculum experts in accordance with the school curriculum, were used. To prevent testing effects and reduce measurement bias, parallel equivalent forms of the Mathematics and Social Studies quizzes were developed. Two independent subject specialists reviewed and validated the equivalence of content, difficulty level, and alignment with curriculum standards. Pre-test and post-test forms were counterbalanced across participants. The same quiz was administered both before and after the physical activity to measure the acute effect. Internal consistency reliability of each quiz form was evaluated (KR-20 ranging from 0.79 to 0.83).

#### 2.3.3. Focus and Task Monitoring Assessment Form

Replicating the validated observational framework of Ridgway et al. (2003), the researchers implemented a structured focus and task-monitoring instrument that generated precise, minute-by-minute behavioral coding of pre-defined positive and negative indicators to quantify students’ on-task duration during 15-min classroom tasks. Observers were fully blinded to group allocation, did not witness intervention delivery, and used anonymized numeric codes for students. Inter-observer reliability (Cohen’s kappa) ranged from 0.82 to 0.91 across training and actual observations. This inter-observer reliability established that the targeted behaviors were measured accurately and consistently according to the proposed procedures. Observations occurred twice weekly over four weeks, capturing both pre- and post-BB intervals in the experimental group and equivalent times in the control group.

#### 2.3.4. Brain Breaks Physical Activity Videos

Videos developed by HOPSports (2017), lasting 3–5 min and containing dance, coordination, and movement activities, were used. The selection was balanced, including highly active, moderately active, and less active videos. Video selection followed a predefined protocol to ensure standardized exposure across participants. A session log was maintained to document adherence, duration, and completion of each session. Intervention fidelity exceeded 95%, and no deviations from the planned protocol were recorded.

### 2.4. Procedure

The intervention spanned four weeks, with the experimental group receiving 48 BB sessions (Application twice randomly on different days of the week for every six lesson hours per day). Sessions used a standardized video selection protocol (balancing high-, moderate-, and low-intensity activities) with >95% fidelity. Each session consisted of three sections (Table 1). Acute academic effects were assessed only in the experimental group during the final week: a 10–12 item quiz (pre-BB) → 10-min high-energy BB video → same quiz re-administered (post-BB). The same teacher delivered instruction to both groups to control for teacher effects. Informal teacher and student feedback was documented separately and not included in primary quantitative analyses. Table 1 displays intervention structure.

### 2.5. Data Analysis

The collected data were analyzed using IBM SPSS Statistics 25.0. Prior to inferential analyses, all variables were screened for missing data, normality, and homogeneity of variance. Missing data were minimal (<3%) and handled using listwise deletion. Assumptions of normality and homogeneity of variance were met for all analyses. For repeated-measures analyses, the assumption of sphericity was satisfied.

To assess baseline equivalence between the experimental and control groups, an independent samples *t*-test was conducted on pre-test attentional control scores. Changes in attentional control were examined using a 2 (Time: pre-test, post-test) × 2 (Group: experimental, control) repeated-measures ANOVA, with particular emphasis on the Time × Group interaction effect. Minute-by-minute on-task behavior data were analyzed descriptively using percentage distributions and visualized through line graphs to illustrate temporal patterns across lessons. Given the observational structure and sample size, these data were not subjected to inferential time-series modeling. Academic achievement (Mathematics and Social Studies quiz scores) within the experimental group was analyzed using paired samples *t*-tests to examine pre- and post-intervention differences. The level of statistical significance was set at α = 0.05 for all analyses. Exact *p*-values, degrees of freedom, and effect sizes were reported consistently across analyses. Effect sizes were calculated using Cohen’s d for *t*-tests and partial eta squared (ηp^2^) for ANOVA. Statistical results were reported in accordance with APA guidelines, including exact *p*-values, degrees of freedom, and effect sizes (Cohen’s d and partial η^2^).

### 2.6. Ethical Considerations

Prior to the research, necessary approvals were obtained from Afyon Kocatepe University Ethics Board (2020/52/02). Additionally, informed consent was obtained from the school administration, classroom teachers, and the parents/guardians of the students. Participation was based entirely on voluntariness, and students were informed of their right to withdraw at any time.

## 3. Results

### 3.1. Attentional Control Levels of the Experimental and Control Groups

As presented in Table 2, there was no statistically significant difference between the experimental and control groups in pre-test attentional control scores, t(47) = −0.55, *p* = 0.583, indicating that the groups were comparable at baseline prior to the intervention.

The repeated-measures ANOVA revealed a significant main effect of time, F(1,47) = 4.37, *p* = 0.042, partial η^2^ = 0.085, suggesting an overall increase in attentional control scores from pre-test to post-test across participants. More importantly, a significant Time × Group interaction was found, F(1,47) = 6.94, *p* = 0.011, partial η^2^ = 0.129, indicating that the change over time differed significantly between groups.

Descriptive statistics showed that attentional control scores increased in the experimental group (from M = 2.27 to M = 2.48), whereas the control group showed a slight decrease (from M = 2.31 to M = 2.28). The between-group main effect was not statistically significant, F(1,47) = 2.48, *p* = 0.122, partial η^2^ = 0.050. These findings suggest that the BB intervention had a positive effect on students’ attentional control over time.

### 3.2. Findings on the Experimental Group’s Mathematics and Social Studies Achievement Tests

The acute effect of the BB activity on quiz performance was measured during the final week of the intervention.

As presented in Table 3, paired-samples *t*-test results indicated no statistically significant difference in Mathematics scores between pre- and post-BB conditions, t(23) = −0.26, *p* = 0.800, d = 0.05. This finding suggests that the BB intervention did not produce a measurable acute effect on Mathematics performance.

In contrast, a statistically significant increase was observed in Social Studies scores following the BB activity, t(23) = −2.26, *p* = 0.033, d = 0.46. Descriptively, mean scores increased from M = 74.45 (SD = 19.10) to M = 79.20 (SD = 15.67). This result indicates a moderate effect size and suggests that the BB activity may have contributed to improved short-term performance in Social Studies.

### 3.3. Findings on the Experimental Group’s On-Task Focus Durations

**Figure 1:**
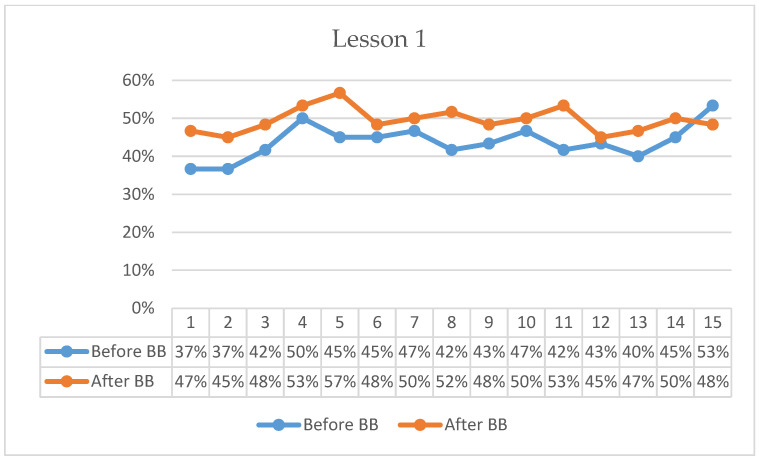
Percentage analysis of students’ on-task focus during Lesson 1.

**Figure 2:**
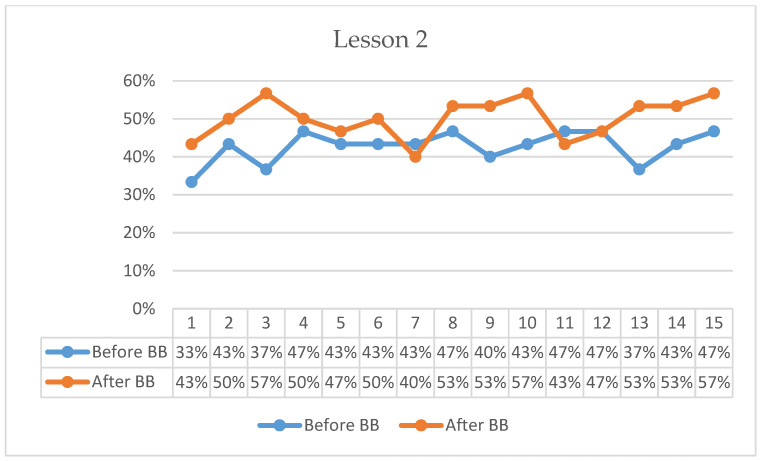
Percentage analysis of students’ on-task focus during Lesson 2.

**Figure 3:**
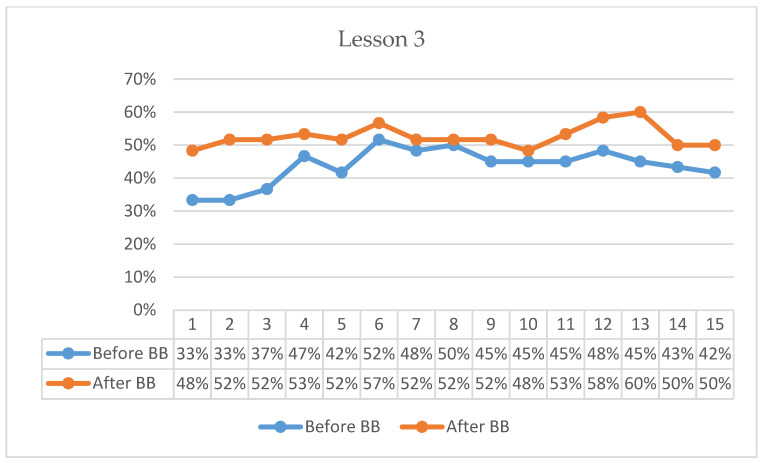
Percentage analysis of students’ on-task focus during Lesson 3.

**Figure 4:**
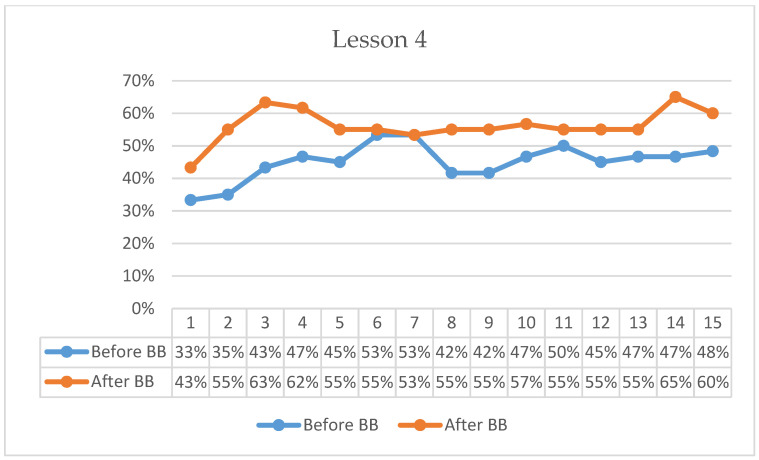
Percentage analysis of students’ on-task focus during Lesson 4.

**Figure 5:**
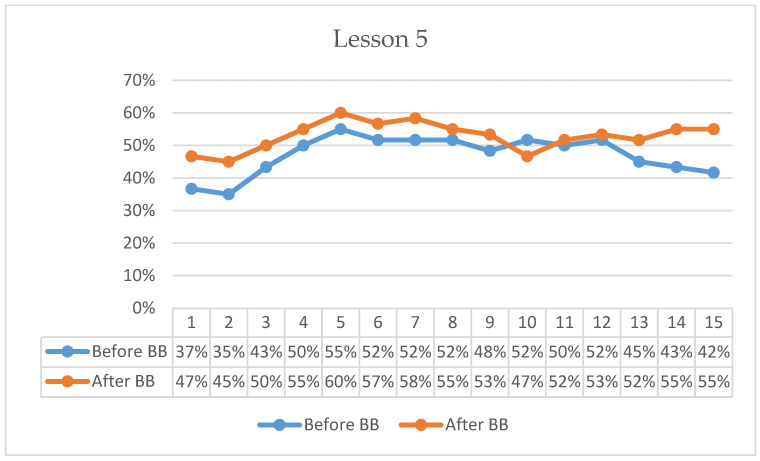
Percentage analysis of students’ on-task focus during Lesson 5.

**Figure 6:**
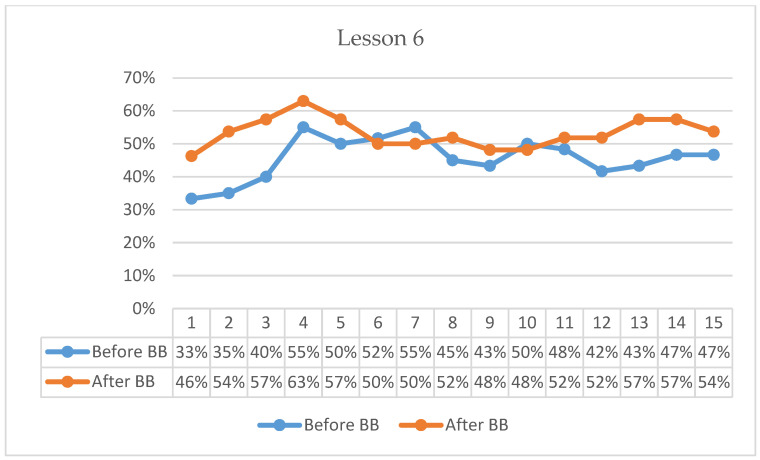
Percentage analysis of students’ on-task focus during Lesson 6.

Figure 1, Figure 2, Figure 3, Figure 4, Figure 5 and Figure 6 present minute-by-minute descriptive patterns of students’ on-task behavior across six lessons. These figures are intended to illustrate temporal engagement patterns before and after BB sessions rather than inferential comparisons. A consistent pattern across all lessons is described below.

In Lesson 1, pre-BB on-task percentages began at 37%, increased to 50% by the 4th minute, gradually declined to 45% by the 14th minute, and rose again to 53% in the final minute (Figure 1). Post-BB on-task percentages started higher at 47%, peaked at 57% by the 5th minute, and gradually decreased to 48% by the 15th minute. Descriptively, the first-minute on-task percentage was 10 percentage points higher following BB compared with the pre-BB observation. Overall, post-BB observations indicated a stronger initial engagement pattern during the early minutes of the lesson.

In Lesson 2, pre-BB on-task percentages began at 33%, increased to 47% by the 4th minute, and ended at 47% in the 15th minute following minor fluctuations (Figure 2). Post-BB on-task percentages started at 43%, rose to 57% by the 3rd minute, and remained at 57% at the end of the 15-min observation period despite gradual fluctuations. Descriptively, the first-minute on-task percentage was 10 percentage points higher following BB compared with the pre-BB observation. Overall, post-BB observations suggested higher initial engagement and more sustained on-task behavior throughout the lesson period.

In Lesson 3, pre-BB on-task percentages began at 33%, increased to 52% by the 6th minute, and declined gradually to 42% by the end of the 15-min observation period (Figure 3). Post-BB on-task percentages started higher at 48%, increased to 57% by the 6th minute, reached 60% in the 13th minute following minor fluctuations, and concluded at 50% in the 15th minute. Descriptively, the first-minute on-task percentage was 15 percentage points higher following BB compared with the pre-BB observation. Overall, post-BB observations indicated stronger initial engagement and generally higher on-task percentages across the lesson period.

In Lesson 4, pre-BB on-task percentages began at 33%, increased to 53% by the 6th minute, and gradually declined to 48% by the end of the 15-min observation period (Figure 4). Post-BB on-task percentages started at 43%, rose to 63% by the 3rd minute, and remained comparatively high, ending at 60% in the 15th minute. Descriptively, the first-minute on-task percentage was 10 percentage points higher following BB compared with the pre-BB observation. Overall, post-BB observations suggested both stronger initial engagement and more sustained on-task behavior throughout the lesson period.

In Lesson 5, pre-BB on-task percentages began at 37%, increased to 55% by the 5th minute, and gradually declined to 42% by the end of the 15-min observation period (Figure 5). Post-BB on-task percentages started at 47%, rose to 60% by the 5th minute, and remained relatively higher, ending at 55% in the 15th minute. Descriptively, the first-minute on-task percentage was 10 percentage points higher following BB compared with the pre-BB observation. Overall, post-BB observations indicated stronger initial engagement and higher on-task percentages across most of the lesson duration.

In Lesson 6, pre-BB on-task percentages began at 33%, increased to 55% by the 4th minute, and gradually declined to 47% by the end of the 15-min observation period (Figure 6). Post-BB on-task percentages started higher at 46%, rose to 63% by the 4th minute, and remained relatively elevated, concluding at 54% in the 15th minute. Descriptively, the first-minute on-task percentage was 13 percentage points higher following BB compared with the pre-BB observation. Overall, post-BB observations indicated stronger initial engagement and generally higher on-task percentages across the lesson period.

Across the six observed lessons, students demonstrated a consistent tendency to begin classroom tasks with higher on-task behavior following BB sessions compared with pre-BB observations. Descriptive averages indicated that first-minute on-task behavior was approximately 12% higher after BB (46%) than before BB (34%). In addition, post-BB observations generally maintained higher on-task percentages throughout the 15-min monitoring period, suggesting that students re-engaged with classroom tasks more rapidly following the activity break.

## 4. Discussion

The present study examined the effects of Brain Breaks (BB) physical activity (PA) videos on fourth-grade students’ attentional control, on-task behavior, and short-term academic achievement. The findings extend previous research by providing a more detailed account of how classroom-based physical activity influences both immediate engagement and attentional processes over time.

A key finding of the study was the significant improvement in attentional control in the experimental group compared to the control group. This result suggests that repeated exposure to BB activities may contribute to the development of students’ attentional regulation. Unlike the immediate patterns observed in on-task behavior, attentional control reflects a broader and more sustained self-regulatory capacity. Therefore, the observed improvement may indicate cumulative benefits of regular participation in short PA breaks. These findings align with previous research demonstrating that classroom-based PA can support executive functioning and attentional processes (Mullender-Wijnsma et al., 2015; Grieco et al., 2016). The underlying mechanism may be explained by short-term increases in physiological arousal and cognitive activation following physical activity, which enhance readiness to focus and regulate attention (Pontifex et al., 2014).

The findings related to academic achievement revealed a differentiated pattern across subject areas. While no significant change was observed in Mathematics scores, Social Studies performance improved following the BB intervention. This divergence suggests that the academic impact of BB activities may depend on the cognitive demands of the subject. Mathematics tasks often require sustained attention, working memory, and sequential problem-solving, which may not be immediately influenced by short-term increases in arousal. In contrast, Social Studies assessments may rely more on reading comprehension, recall, and general attentional readiness, making them more responsive to the immediate effects of physical activity. This pattern is consistent with prior research indicating that movement-based interventions may differentially affect academic domains depending on task characteristics (Wold et al., 2023; López et al., 2020). These findings highlight the importance of considering subject-specific factors when evaluating the educational impact of PA interventions.

One of the most important contributions of this study lies in its minute-by-minute analysis of students’ on-task behavior across multiple classroom sessions. The findings revealed a consistent pattern across all six lessons, with higher initial on-task behavior observed following BB sessions, particularly within the first 1–5 min. This “initial engagement effect” suggests that students are able to re-engage with classroom tasks more rapidly after participating in short physical activity breaks. Importantly, this effect was observed consistently across sessions, supporting its robustness beyond a single observation.

This temporal pattern provides new insight into how the effects of PA unfold within classroom settings. While previous studies have primarily focused on overall changes in attention, the present findings indicate that the benefits of BB may be most pronounced during the early phase of task engagement. This suggests that BB activities may function as a short-term regulatory mechanism that facilitates transitions into learning rather than sustaining attention uniformly over time. The observed pattern may be explained by transient increases in arousal and cognitive activation following PA, which enhance immediate readiness to engage but gradually diminish over time.

The findings related to on-task behavior are consistent with research demonstrating that short PA breaks can improve classroom engagement and reduce off-task behavior (Mavilidi et al., 2019; Norris et al., 2018). In particular, cognitively engaging physical activities have been shown to be effective in enhancing selective attention and behavioral regulation (Heemskerk et al., 2019). These findings support the notion that not only the presence of movement, but also the nature of the activity, may influence its effectiveness in promoting classroom engagement.

Taken together, the results suggest that BB activities may influence students’ learning processes through multiple pathways. On the one hand, they appear to produce immediate improvements in behavioral engagement, as reflected in higher initial on-task behavior. On the other hand, repeated exposure may contribute to the development of attentional control over time. This dual effect highlights the potential of BB as both an immediate and cumulative intervention within classroom settings.

From a practical perspective, these findings suggest that BB activities may be most effective when strategically implemented at key transition points during instruction, such as at the beginning of lessons or following periods of sustained sedentary activity. By enhancing students’ readiness to engage, BB may help optimize the use of instructional time without requiring major changes to the curriculum.

Several limitations should be acknowledged. First, the study was conducted with a relatively small convenience sample drawn from a single private school, which may limit the generalizability of the findings. The participants were primarily from middle- to upper-socioeconomic backgrounds, which further restricts the representativeness of the sample. Second, although the minute-by-minute observations provided detailed insight into classroom engagement, these data were analyzed descriptively. Future research may benefit from applying longitudinal or multilevel analytical approaches to more rigorously examine temporal patterns in on-task behavior. Finally, academic achievement was assessed using short-term quizzes, which may not fully capture longer-term learning outcomes.

Future studies should explore how different types, durations, and intensities of physical activity influence both attentional processes and academic outcomes. In particular, further research is needed to examine how PA interventions can be optimally integrated into different subject areas to maximize their educational impact.

## 5. Conclusions

The present study provides evidence that Brain Breaks (BB) physical activity (PA) videos may support both immediate classroom engagement and attentional processes in elementary school students. A key contribution of this study is the identification of a consistent “initial engagement effect,” demonstrating that the impact of BB is particularly pronounced within the first few minutes of classroom task engagement. Descriptive findings indicated higher initial on-task behavior following BB sessions, suggesting improved readiness to engage in learning tasks. In addition, attentional control improved over time in the experimental group, indicating potential cumulative benefits of repeated BB exposure. The findings also suggest that the academic effects of BB may be subject-specific, with improvements observed in Social Studies but not in Mathematics. These differences may be explained by the varying cognitive demands of subject areas, as well as short-term increases in physiological arousal and cognitive activation following PA. Overall, BB activities may represent a practical and low-cost strategy to support students’ readiness for learning, particularly in classroom contexts characterized by prolonged sedentary behavior.

## Figures and Tables

**Table 1:** Intervention structure.

Section	Time	Focus
One	15 min	Task assignment/instruction related to the lesson.
Two	10 min	BB PA video session.
Three	15 min	Task assignment/instruction related to the lesson.

**Table 2:** Descriptive statistics, baseline comparison, and repeated-measures ANOVA results for attentional control scores.

Variable	Group	N	Pre-Test M ± SD	Post-Test M ± SD	Baseline Comparison t(df), *p*	Time Effect F(df1,df2), *p*, ηp^2^	Time × Group Effect F(df1,df2), *p*, ηp^2^	Group Effect F(df1,df2), *p*, ηp^2^
Attentional Control	Experimental	24	2.27 ± 0.22	2.48 ± 0.30	t(47) = −0.55, *p* = 0.583	F(1,47) = 4.37, *p* = 0.042, 0.085	F(1,47) = 6.94, *p* = 0.011, 0.129	F(1,47) = 2.48, *p* = 0.122, 0.050
	Control	25	2.31 ± 0.19	2.28 ± 0.24				

Note. M = Mean; SD = Standard Deviation; ηp^2^ = partial eta squared.

**Table 3:** Paired samples *t*-test results for the experimental group’s Mathematics and Social Studies quiz scores.

Variable	Time	N	M ± SD	t(df)	*p*	Cohen’s d
Mathematics	Pre-BB	24	73.04 ± 14.53	−0.26 (23)	0.800	0.06
	Post-BB	24	73.43 ± 14.01			
Social Sciences	Pre-BB	24	74.45 ± 19.10	−2.26 (23)	0.033 *	0.46
	Post-BB	24	79.20 ± 15.67			

* *p* < 0.05, Pre-BB: Pre-Brain Breaks, Post-BB: Post-Brain Breaks.

## Data Availability

The raw data supporting the conclusions of this article will be made available by the authors on request.

## Abbreviations

The following abbreviations are used in this manuscript:

PA	Physical Activity
ACS-C	the Attentional Control Scale for Children
BB	Brain Breaks
